# Rod Driven Frequency Entrainment and Resonance Phenomena

**DOI:** 10.3389/fnhum.2016.00413

**Published:** 2016-08-18

**Authors:** Christina Salchow, Daniel Strohmeier, Sascha Klee, Dunja Jannek, Karin Schiecke, Herbert Witte, Arye Nehorai, Jens Haueisen

**Affiliations:** ^1^Institute of Biomedical Engineering and Informatics, Technische Universität IlmenauIlmenau, Germany; ^2^Institute of Medical Statistics, Computer Sciences and Documentation, Jena University Hospital, Friedrich Schiller University JenaJena, Germany; ^3^Preston M. Green Department of Electrical and Systems Engineering, Washington University in St. Louis, St. LouisMO, USA; ^4^Hans Berger Department of Neurology, Biomagnetic Center, Jena University Hospital, Friedrich Schiller University JenaJena, Germany

**Keywords:** visual system, alpha rhythm, photic driving, resonance phenomena, frequency entrainment, magnetoencephalography, rod-driven

## Abstract

A controversy exists on photic driving in the human visual cortex evoked by intermittent photic stimulation. Frequency entrainment and resonance phenomena are reported for frequencies higher than 12 Hz in some studies while missing in others. We hypothesized that this might be due to different experimental conditions, since both high and low intensity light stimulation were used. However, most studies do not report radiometric measurements, which makes it impossible to categorize the stimulation according to photopic, mesopic, and scotopic vision. Low intensity light stimulation might lead to scotopic vision, where rod perception dominates. In this study, we investigated photic driving for rod-dominated visual input under scotopic conditions. Twelve healthy volunteers were stimulated with low intensity light flashes at 20 stimulation frequencies, leading to rod activation only. The frequencies were multiples of the individual alpha frequency (α) of each volunteer in the range from 0.40 to 2.30^∗^α. Three hundred and six-channel whole head magnetoencephalography recordings were analyzed in time, frequency, and spatiotemporal domains with the Topographic Matching Pursuit algorithm. We found resonance phenomena and frequency entrainment for stimulations at or close to the individual alpha frequency (0.90–1.10^∗^α) and half of the alpha frequency (0.40–0.55^∗^α). No signs of resonance and frequency entrainment phenomena were revealed around 2.00^∗^α. Instead, on-responses at the beginning and off-responses at the end of each stimulation train were observed for the first time in a photic driving experiment at frequencies of 1.30–2.30^∗^α, indicating that the flicker fusion threshold was reached. All results, the resonance and entrainment as well as the fusion effects, provide evidence for rod-dominated photic driving in the visual cortex.

## Introduction

Intermittent photic stimulation (IPS) is an important method in neurophysiological examinations in clinical practice. In research, it has been widely used to explore basic mechanisms of bioelectrical rhythmic activity in the human brain (Lazarev et al., 2001; Stough et al., 2001; Takahashi, 2005). Photic driving can be induced by IPS, and is characterized by rhythmic brain activity related to that of the stimulus (Van der Tweel and Verduyn Lunel, 1964; Regan, 1965; Lazarev et al., 2001). Two main measureable phenomena in electroencephalography (EEG) and magnetoencephalography (MEG) are observed: frequency entrainment and resonance effects. Frequency entrainment is characterized by the frequency-locking of a free running brain rhythm to the stimulation frequency of the IPS (Hayashi, 1985; Silberstein, 1995; Pikovsky et al., 2001). Resonance phenomena occur for the stimulation at or near spontaneous frequencies and its harmonics and are indicated by enlarged response amplitudes (Başar et al., 1997; Herrmann, 2001). In the case of the alpha rhythm, the effects are called alpha frequency entrainment and alpha resonance. The quantization of the photic driving effect is widely used to study differences between healthy brain activity and several neurophysiological diseases, such as schizophrenia, epilepsy, and dementia, e.g., (Drake et al., 1989; Fukami et al., 2008).

The mechanisms causing photic driving and their connection to the alpha rhythm are not fully understood. Gebber et al. (1999) investigated the human alpha rhythm by stimulating with light flashes at 3–12 Hz and observed a frequency-locking between the alpha frequency and the stimulation frequency. They concluded that a non-linear oscillator must generate the alpha rhythm. Herrmann (Herrmann, 2001) performed IPS from 1 to 100 Hz in steps of 1 Hz and found that the evoked responses exposed clear resonance phenomena (amplitude increase) for the stimulation with 10, 20, 40, and 80 Hz. The author suggests that the strong resonance at 10 Hz may relate to mechanisms responsible for the spontaneous alpha activity while the resonance at 40 Hz may reflect neuronal structures producing gamma activity. Miranda de Sá and Infantosi (2005) stimulated with flicker frequencies of 3, 4, 5, 6, 8, 10, 12, and 15 Hz and reported comparatively stronger photic driving in the EEG when stimulating close to the alpha frequency. No frequency entrainment was observed when stimulating with 15 Hz. Kalitzin et al. (2002) performed the first photic driving experiments on the basis of simultaneous EEG/MEG recordings. Further, Schwab et al. (2006) stimulated with flicker frequencies that were adapted to the individual alpha frequency of each volunteer while measuring EEG and MEG. They reported frequency entrainment and a significantly elevated alpha peak in the power spectra when stimulating at or close to the individual alpha frequency (∼0.90–1.10^∗^α) or half the individual alpha frequency (∼0.45–0.55^∗^α) being slightly more pronounced in the MEG compared to the EEG. By means of a topographic analysis with spatiotemporal Matching Pursuit algorithms, Halbleib et al. (2012) examined the spatial stability of photic driving based on the data of Schwab et al. (2006). They quantified stages of engagement and disengagement, where engagement describes the effect of EEG/MEG topography stabilization after the beginning of the stimulation indicating the synchronization of the neural oscillators. Disengagement specifies the effect of topography destabilization after the end of the stimulation and thereby the desynchronization of the neural oscillators. The described effects support the hypothesis of a coupled system of neural oscillators with an individual resonance frequency underlying the photic driving effect, as reported by Fedotchev et al. (1990), Lopes da Silva (1991) and Stam et al. (1999).

While Herrmann (2001) and Lazarev et al. (2001, 2004) reported that flicker stimulation at frequencies higher than 12 Hz led to resonance effects, Schwab et al. (2006) and Halbleib et al. (2012) found neither resonance phenomena nor frequency entrainment at stimulation frequencies higher than 1.10^∗^α. The used stimulation setup could be a reason for the different results, because Herrmann (2001) and Lazarev et al. (2001, 2004) applied high intensity light stimulation; whereas Schwab et al. (2006) and Halbleib et al. (2012) used a combination of relatively low light intensity eyes closed stimulation and a presentation in a dimly illuminated room. The conditions of the latter setup are characteristic for scotopic vision, which is dominated by rod photoreceptors. However, none of the studies reported radiometric measurements for their stimulation setups. For scotopic vision, the existence of two different types of rod pathways and thereby of two temporally different rod signals is postulated (Hess and Nordby, 1986; Sharpe et al., 1989): a slow, sensitive pathway resulting in a slow, sensitive signal superimposed by a fast, insensitive signal from the second pathway. In the sensitive pathway, rod signals travel via rod ON bipolar cells to different ganglion cells. The synaptic mechanism between the cells allows the transmission of single-photon signals and is very effective at low light intensities. In the insensitive pathway, rod signals travel very fast via gap junctions between rod bipolar cells and ganglion cells. As the light intensity level is increased, the slow rod signal changes gradually to the fast rod signal (Stockman et al., 1991). Owing to phase shift effects at approximately 15 Hz, both signals interfere and cancel each other out in this frequency range. However, the processing of rod vision is considerably slower than that of cone vision. Sharpe et al. (1989) reported maximum rod perceptible flicker frequencies between 20 and 25 Hz. Further, Stockman et al. (1995) investigated flicker-evoked slow and fast rod signals using the electroretinogram (ERG) and reported amplitude losses of nearly 90% at an IPS of 18 Hz in comparison to the amplitudes at 8 Hz. It can be summarized that the flicker perception by rods is limited by a flicker fusion threshold of approximately 15 Hz, while cones can perceive frequencies up to 100 Hz (Sharpe et al., 1989; Tovée, 1996). Our hypothesis of a rod-dominated vision in the setup of Schwab et al. (2006) would therefore explain the absence of entrainment and resonance effects when stimulating with flicker frequencies higher than 15 Hz despite they were found in other setups.

The aim of this study is to investigate for the first time photic driving under the condition of scotopic vision in the human visual system. We chose a setup comparable to the study of Schwab et al. (2006) with a low intensity light stimulation at frequencies that were adapted to the individual alpha frequency in the range from 0.40 to 2.30^∗^α. Radiometric measurements allowed us to specifically target scotopic vision. We expected to observe photic driving for the stimulation at and around 0.50^∗^α and 1.00^∗^α, but no effects at and around 2.00^∗^α, since no rod input to the visual system and thus to the cortical neuronal network can be expected above ∼15 Hz. As the MEG reveals a slightly better performance in previous photic driving studies (Schwab et al., 2006), we recorded a 306-channel MEG. The data were analyzed in the time, frequency, and spatiotemporal domains.

In the following, we give definitions of the different aspects of the photic driving phenomenon as they are essential for the subsequent sections:

• ***Photic driving***: can be induced by IPS, and is characterized by rhythmic brain activity related to that of the stimulus; two main measureable phenomena in EEG and MEG are observed: frequency entrainment and resonance effects (Van der Tweel and Verduyn Lunel, 1964; Regan, 1965; Lazarev et al., 2001),
• ***Frequency entrainment***: frequency-locking of a free running brain rhythm to the stimulation frequency of the IPS (Hayashi, 1985; Silberstein, 1995; Pikovsky et al., 2001),
• ***Resonance effects***: enlarged response amplitudes when applying an IPS frequency in the range of a free running brain rhythm or harmonically related frequencies (Başar et al., 1997; Herrmann, 2001),
• ***Engagement***: effect of EEG/MEG topography stabilization after the beginning of the IPS indicating the synchronization of the neural oscillators,
• ***Disengagement***: topography destabilization after the end of the IPS, and thereby the desynchronization of the neural oscillators (Halbleib et al., 2012),
• ***Flicker fusion threshold:*** frequency at which an IPS is perceived as a static illumination.

## Materials and Methods

### Volunteers

Twelve healthy volunteers, six male and six female, between 21 and 42 years of age (median 26) participated in the study. All volunteers reported normal vision and no neurological disorders. Written informed consent was obtained. The study was approved by the Ethics Committee of the Faculty of Medicine of the Friedrich-Schiller-University Jena.

### Stimulation and Recording

The participants were stimulated, eyes closed, by intermittent flickering light at 20 different stimulation frequencies that were multiples of the subject’s individual alpha frequency α in the range from 0.40 to 2.30 times α. The stimulation frequencies of 0.40, 0.45, 0.50, 0.55, 0.60, 0.70, 0.80, 0.90, 0.95, 1.00, 1.05, 1.10, 1.30, 1.70, 1.90, 1.95, 2.00, 2.05, 2.10, and 2.30^∗^α, were presented in a randomized order and the order of presentation was reversed for half of the volunteers. The stimuli were produced by two light-emitting diodes. Optical fibers transmitted the stimuli from outside the recording room to light diffusers located approximately 10 cm in front of the volunteer. To determine the effective eyelid luminance of the closed eye, we performed a radiometric measurement. Therefore, a research radiometer (IL1700, International Light Technologies, Peabody, MA, USA) was placed equivalent to the position of the subject’s eye. Using the eyelid transmission of Bierman et al. (2011) and the assumption that transmitted light through the eyelid follows a Lambertian angular distribution, we computed a luminance of 0.0003 cd/m^2^. According to the work of Stockman and Sharpe (2006) this leads to scotopic vision. The volunteers were placed in seating position in a magnetically shielded room, where a whole head MEG was recorded using 102 magnetometers and 204 planar gradiometers, arranged in 102 sensor triplets (Vectorview; Elekta Neuromag Oy, Helsinki, Finland).

**FIGURE 1:**
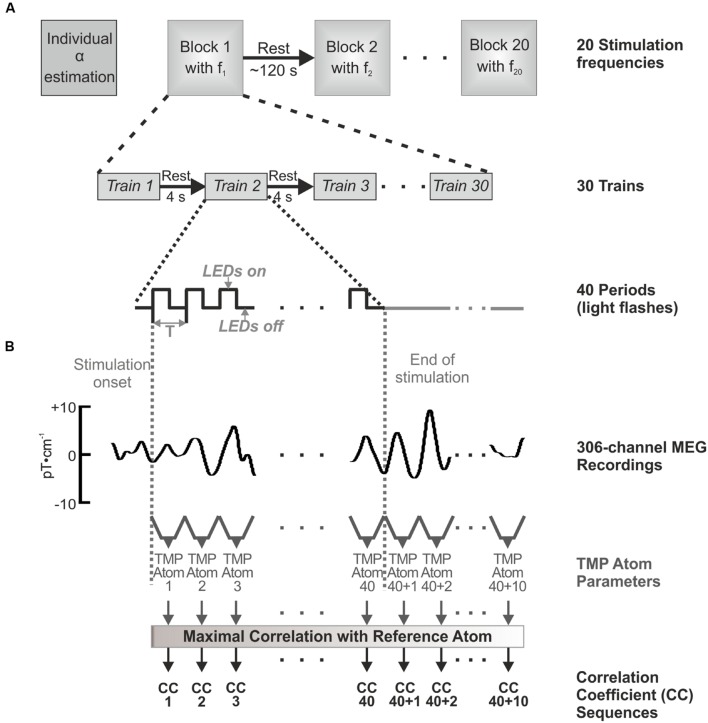
**Experimental setup **(A)** and visualization of the Topographic Matching Pursuit (TMP) approach **(B)**.** **(A)** For each volunteer, twenty stimulation frequencies were applied in a randomized order, where for each frequency 30 trains of 40 flashes were presented. The trains were averaged in the data processing. **(B)** Those averaged trains were then analyzed using TMP. One TMP atom was calculated for each stimulus and for the duration of 10 periods after the offset of the IPS. For each volunteer, those TMP atoms were compared with the individual reference atom. The maximum correlation coefficient of this comparison was extracted for each atom (40 + 10), which formed the correlation coefficient sequences.

At the beginning of each measurement, MEG during resting state (mind rest) with closed eyes (60 s) was recorded to estimate the individual alpha rhythm of each volunteer via determination of the peak frequency in the amplitude spectrum. As the light in the chamber was dimmed, rod-dominated vision was assumed after an adaption time of 8–10 min. Each stimulation frequency was presented in one frequency block consisting of 30 trains of 40 periods, which correspond to 40 light flashes as illustrated in **Figure 1A.** Resting periods of 4 s between the trains and of approximately 120 s between the frequency blocks were recorded. After presenting ten stimulation frequencies, the recording was paused for 5–10 min to allow a short recovery of the volunteer before presenting the following 10 frequency blocks. The experiment lasted approximately 90 min plus preparation time for each volunteer. The MEG was sampled with 1000 Hz, and hardware filtered between 0.1 and 300 Hz.

### Data Analysis

#### Data Processing

The data were digitally bandpass filtered between 2 and 30 Hz with a zerophase Butterworth filter of order 4, and a baseline correction on the data of each stimulation frequency as well as a linear trend elimination were applied. The recordings were analyzed using an average over 30 trains for each stimulation frequency. Signal processing was performed using MATLAB version 7.9.0 (The Mathworks Inc., Natick, MA, USA), and statistical analyses were performed with the statistical software package SPSS 21.0.0.0 (IBM, Armonk, NY, USA).

#### Frequency Domain

Amplitude and power spectra were calculated via fast Fourier transform (FFT) for each stimulation frequency (interval from stimulus 1–40) and resting alpha (time frame as for 1.00^∗^α), averaged over 24 occipital gradiometer channels, covering the back part of the head. The spectral power peak at the stimulation frequency was determined for each volunteer and each presented frequency.

For the resting state measurement and every stimulation frequency measurement of every volunteer, the peak amplitude at the identified alpha frequency was determined in the spectra. Following the analysis concept of Fukami et al. (2008), we computed the ratio between the alpha peak amplitude during stimulation and the resting state alpha peak amplitude for each volunteer and each stimulation frequency to investigate the amount of alpha activity for each IPS frequency. We refer to the resulting values as *alpha peak ratios*. Those ratios were further tested for statistically significant differences by means of a MANOVA and *post hoc* paired *t*-tests (significance level 0.05). All statistical tests in this paper are based on the whole study population, unless stated otherwise. The following null hypothesis *H*_0_ was tested on the *alpha peak ratios* for each stimulation frequency via paired *t*-tests: the distribution of the *alpha peak ratios* has a mean of one, μ = 1, stating that the alpha activity is equal with or without stimulation. As alternative hypothesis, both options were tested, μ > 1 and μ < 1.

Moreover, the highest peak amplitude in each spectrum was determined in the range of the stimulation frequency (±2 Hz). We refer to this peak as *response frequency*. We then defined two conditions to classify an alpha frequency entrainment. Firstly, entrainment means that the determined response frequency is exactly at the stimulation frequency or at its harmonics. Secondly, we specified an amplitude threshold for the determination of the response frequency to prevent an incorrect classification of the response due to noise and interindividual variability. The amplitude of the response peak in the spectrum had to exceed 20% of the maximum frequency peak that was found throughout the whole study population.

#### Spatiotemporal Domain

Engagement and disengagement in the spatiotemporal domain were investigated by performing a mapping analysis using Topographic Matching Pursuit (TMP). TMP was first introduced by Gratkowski et al. (2007) as an extension on Matching Pursuit (Mallat and Zhang, 1993) for the purpose of multichannel analysis such as EEG and MEG data. TMP allows for a dimension reduction of the spatiotemporally distributed data by using extended Gabor atoms, so-called *TMP atoms g*_γ_, for approximation. *TMP atoms* consist of modulated and scaled Gaussian window functions. They describe a signal completely in the time, frequency, and spatial domain for a specified period of time by using only one parameter vector γ consisting of five parameters. γ is a function of the scale *s*, translation *u*, and modulation ξ. Two additional parameters hold a list of amplitudes and a list of phase shifts that adjust the atom for each channel. The values of *s*, *u*, and ξ are chosen that way that the picked atom shows the highest correlation over all channels and therefore provides topographic information. TMP was especially suitable for our aim of analyzing oscillations due to the use of time-frequency dictionaries (Mallat and Zhang, 1993; Gratkowski et al., 2007). Furthermore, TMP allows an evaluation of the topographic relationships in photic driving based on atom parameters, especially *s*, *u*, and ξ, rather than just based on visual observations of topographies.

*TMP atom* sets were calculated consisting of one *TMP atom* for the period of each visual stimulus (1–40) and for the ten periods after the end of the stimulation due to the expected reverberation (preservation) of the alpha rhythm (Sakamoto et al., 1993). *TMP atoms* were thereby computed separately for the 40 + 10 periods of every stimulation frequency and every volunteer. A so-called individual reference atom was calculated for every volunteer by averaging the 40 *TMP atoms* at the stimulation with 1.00^∗^α. This reference atom was supposed to represent a strong alpha activity in photic driving. Normalized correlation coefficients between the reference atom and the 40 + 10 TMP atoms were calculated for each channel, stimulation frequency, and volunteer (see **Figure 1B**). The maximum values were used to form so-called correlation coefficient sequences consisting of 40 + 10 coefficients for each channel, stimulation frequency, and volunteer. Those values were taken as a measure for the similarity between the reference atom (and thereby photic driving effect) and the individual response approximated in the *TMP atoms.* For a more detailed description of the procedure, please see (Gratkowski et al., 2007) and (Halbleib et al., 2012). The correlation coefficient sequences as well as the TMP parameters *s*, *u*, and ξ were visually and statistically evaluated.

#### Time Domain

If the flicker fusion threshold of rod perception was reached by the flicker stimulus in our experimental setup, the volunteer would perceive the IPS as constant light stimulation. At the beginning and the end of continuous visual stimulation, an on-response shortly after the beginning as well as an off-response shortly after the end of the stimulation can be expected (Clynes et al., 1964). To investigate possible on-responses and off-responses in our data, the time signal was considered. Additionally, the mean envelope of each stimulation frequency block was calculated over all channels using the Hilbert transform for the signal 500 ms before the stimulation onset until 3 s after the end of the stimulation. *On-responses* were defined as the first peak in the envelope arising after the onset of the flicker stimulus, *off-responses* as the first outstanding peak after the end of the IPS. The peak amplitude and the latency of the peak maximum were determined for both responses in each stimulation block of each volunteer. The occurrence and parameters of on-response and off-response were tested statistically by means of an analysis of variances (ANOVA; significance level 0.05) and *post hoc* paired *t*-tests. The relevant null hypotheses and alternative hypotheses are reported in the section “Results”.

## Results

### Overview

**FIGURE 2:**
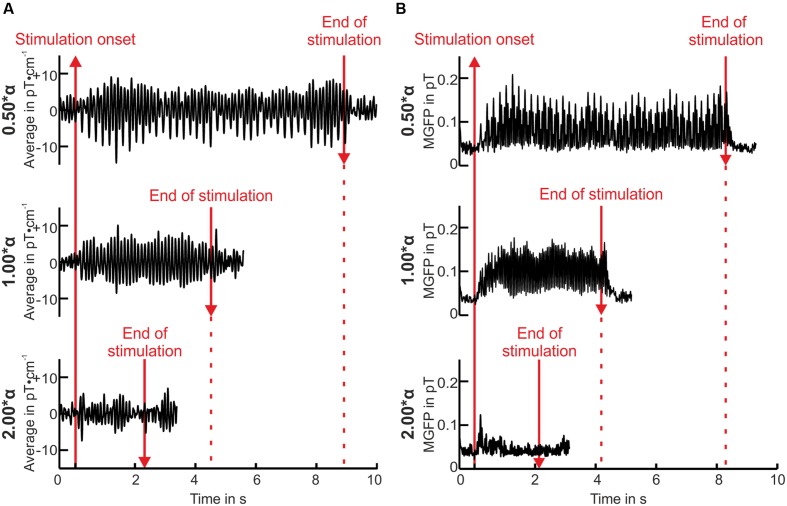
**Averaged single right-occipital gradiometer channel measurement data in pT⋅cm^-1^ over time **(A)** and square root of mean global field power (MGFP) of all magnetometers (pT) over time **(B)** of volunteer P01.** Baseline is displayed for 500 ms before and 1 s after the end of the stimulation (see red lines).

The mean resting state alpha frequency over all volunteers was 10.58 ± 1.14 Hz. An example of the preprocessed averaged data of one volunteer is illustrated in **Figure 2A** for three stimulation frequencies. Substantial amplitude rises indicating strong resonance effects were found for a stimulation at the individual alpha frequency (1.00^∗^α), and at half the individual alpha frequency (0.50^∗^α). The response was preserved shortly after the end of the stimulation. By contrast, stimulation at twice the individual alpha frequency (2.00^∗^α) did not evoke a highly increased oscillatory response. This impression was reinforced considering the square root of mean global field power (MGFP) over all magnetometer channels, exemplarily displayed in **Figure 2B.** The here presented course of the square root of the MGFP was representative for 83% of the volunteers. The remaining volunteers showed weaker responses toward the IPS with small amplitudes.

### Frequency Domain

**FIGURE 3:**
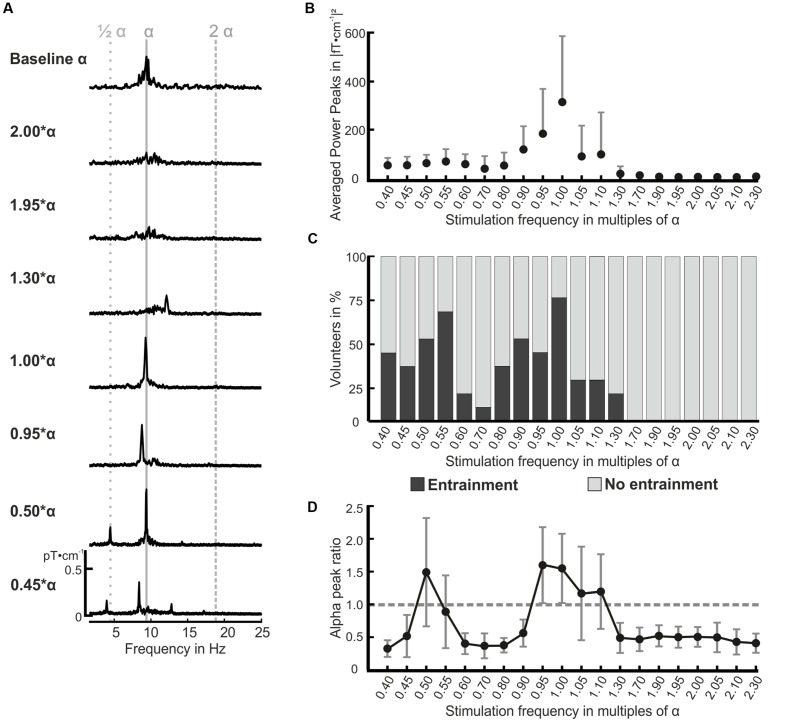
**Amplitude spectra for seven stimulation frequencies and resting MEG (resting state α) of volunteer P01 **(A)**, mean power for all volunteers and all stimulation frequencies **(B)**, frequency entrainment classification for all volunteers **(C)**, and the mean alpha peak ratio over all volunteers **(D)**.** **(A)** The shown amplitude spectra (pT⋅cm^-1^) were calculated over 24 occipital gradiometer channels and are displayed in the frequency range from 2 to 25 Hz. The estimated individual alpha frequency of the presented volunteer in **(A)** was 9.6 Hz. Resonance phenomena become obvious for the stimulation frequencies 1.00, 0.95, 0.50, and 0.45^∗^α. Please see Supplementary Material for amplitude spectra of all volunteers. **(B)** Presents the mean and positive standard deviation power peaks for all volunteers and stimulation frequencies. **(C)** The volunteer’s responses in terms of frequency entrainment were classified for each stimulation frequency (*x*-axis) according to our definition of entrainment and no entrainment in the FFT spectra (concurrence and amplitude threshold). One hundred percent on the ordinate equal 12 volunteers. **(D)** The alpha peak ratio between the alpha peak of each stimulation frequency and the resting state alpha peak is displayed for all 20 stimulation frequencies (*x*-axis) as mean and standard deviation for all volunteers. The dotted gray line marks the alpha peak ratio value of 1: all values higher than this indicate an increased alpha activity.

Exemplary spectra of one volunteer are shown in **Figure 3A** (see Supplementary Material for spectra of all volunteers). The peaks in the spectra demonstrate the photic driving effect for the stimulation frequencies 0.45, 0.50, 0.95, 1.00, as well as 1.30^∗^α. On the contrary, the photic driving was absent for the stimulation with 1.95 and 2.00^∗^α in this volunteer, which is indicated by the missing peaks in the spectra at these two frequencies. At 0.45 and at 0.95^∗^α, the characteristic frequency entrainment can be observed, which is indicated by the peaks at the stimulation frequency and a missing peak at 0.50 and at 1.00^∗^α, respectively. **Figure 3B** displays the spectral power of the stimulation frequencies for all volunteers. On average, the power for IPS between 1.70 and 2.30^∗^α was no more than 3.58 (fT⋅cm^-1^)^2^. The highest power peaks were found for IPS close to the individual alpha frequency with a maximum of 312.5 (fT⋅cm^-1^)^2^ for the stimulation with 1.00^∗^α. The stimulation with IPS between 0.40 and 0.80^∗^α revealed power peaks between 35.8 and 45.7 (fT⋅cm^-1^)^2^, indicating enhanced rhythmic activity at those stimulation frequencies.

The occurrence of frequency entrainment varied strongly between the volunteers. The responses were classified into two groups for each stimulation frequency as shown in **Figure 3C**: entrainment, and no entrainment (see “Frequency Domain” for the classification rules). We found entrainment in the flicker frequency range from 0.40 to 1.30^∗^α. In our population, frequency entrainment occurred most frequently for stimulation frequencies close to the individual alpha frequency and half of the individual alpha frequency. On average, 7 out of 12 volunteers showed frequency entrainment effects for the stimulation with 0.40, 0.45, 0.50, 0.55, 0.90, 0.95, 1.05, and 1.10^∗^α. No entrainment was found for stimulation frequencies higher than 1.30^∗^α.

The strong oscillatory responses in the alpha band at stimulations with the individual alpha frequency and its first subharmonic were underlined by the calculated alpha peak power ratios depicted in **Figure 3D** (average over all volunteers). We found ratios with a value larger than 1.0, indicating an increased activity in the alpha band, for the stimulation at 0.50, 0.95, 1.00, 1.05, and 1.10^∗^α. For the stimulation frequencies 0.40, 0.45, 0.60–0.90, and 1.30–2.30^∗^α, we found significantly reduced alpha activity by means of a paired *t*-test (*H*_0_: μ = 1; *p* < 0.05) between the alpha peak in the responses and the resting state alpha peak. A statistical comparison of the ratios for each stimulation frequency [*H*_0_: μ_1_(*f*_1_) = μ_2_(*f*_2_); *p* < 0.05] revealed that the ratios at 0.40, 0.45, 0.60–0.90, and 1.30–2.30^∗^α were significantly decreased compared to the ratios of the flicker frequencies around 0.50 and 1.00^∗^α. Interestingly, the calculated ratios showed larger variances for frequencies evoking a resonance phenomenon than for the others as seen in **Figure 3D.**

### Spatiotemporal Domain

**FIGURE 4:**
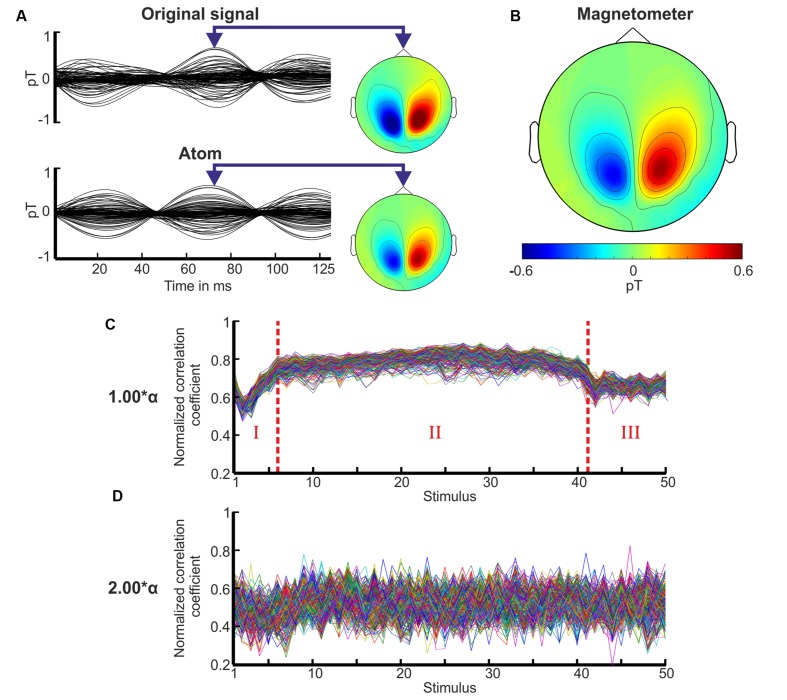
**Approximation, topography of the reference atom, and correlation coefficient sequence of the TMP atoms.** **(A)** The original measurement of the 20th stimulus is shown for the whole period of 125 ms for the stimulation with 1.00^∗^α of volunteer P10. Underneath its approximation by a TMP atom is displayed. The topographies depict the field distributions of the magnetometers using the same scale as in **(B)**. **(B)** The topography of the magnetometers is plotted for the resulting reference atom of volunteer P10 at the same time as in **(A)**. **(C)** The mean correlation coefficient sequence for each channel over all subjects is depicted for the stimulus periods 1–40 and 10 periods after the end of stimulation (*x*-axis) for the flicker stimulation with 1.00^∗^α. The coefficients are normalized in the range from 0 to 1. Three characteristic *phases* are observed marked by dotted red lines and numbers: *phase I* increase, *phase II* plateau, and *phase III* decrease. **(D)** Mean correlation coefficient sequence for each channel over all subjects for the stimulation with 2.00^∗^α over the same stimulus range as in **(C)**.

In general, the TMP atoms approximated both the oscillatory activity and its topographic distribution well as demonstrated in **Figure 4A.** The TMP atom with its five parameters captured all the relevant spatiotemporal features of the original signal. The resulting reference atom of this volunteer is exemplary shown in **Figure 4B** revealing strong activity above the parietal and occipital lobe.

Distinct TMP correlation coefficient sequences exposed three *phases* characterizing the engagement and disengagement process as highlighted in **Figure 4C.** The mean correlation coefficient sequence over all volunteers is displayed for the stimulation with 1.00^∗^α. In *phase I*, the correlation coefficients increased until stimulus 5–10, accompanied by an adjustment of the frequency parameter of the TMP atoms. This frequency parameter increased until stimulus 5–10. This indicated the engagement process: the neural network progressively synchronizes with the IPS. *Phase I* was followed by a stable plateau (*phase II*) in the correlation coefficients as well as in the frequency parameters for those participants who showed a photic driving effect. Shortly after the end of the IPS, a decrease of the correlation coefficients could be observed (*phase III*) indicating the disengagement (desynchronization) process after a short preservation of the resonance state (Sakamoto et al., 1993) for 1–3 periods. The frequency parameters revealed significantly higher variances for *phases I* and *III* than for *phase II* [*t*-test; e.g., *H*_0_: μ_1_(var_1_) = μ_2_(var_2_); *p* < 0.01].

We classified the IPS responses of the volunteers into three groups (good, moderate, and weak responses) based on the establishment of the three *phases* in the correlation coefficient sequences. If all three phases could be identified by eye and the variance of the coefficients was below a predefined threshold (0.004), the response was assigned to be a good response. If all three phases could be identified by eye but the variance of the coefficients exceeded the threshold, the response was assigned to be moderate. If the phases could not be identified by eye, the responses were classified as weak. We found that 50–100% of the volunteers showed good to moderate photic driving responses for the stimulation frequencies around alpha (0.90, 0.95, 1.00, 1.05, and 1.10^∗^α), and 33–42% for an IPS at 0.50 and 0.55^∗^α. The stimulation frequencies 0.45, 0.60, 0.70, and 0.80^∗^α yielded moderate responses in 1–2 volunteers, while the remaining stimulation frequencies yielded only weak responses. For every volunteer, the TMP correlation coefficient sequences presented an unsteady course with the absence of distinct *phases* for all stimulation frequencies from 1.30 to 2.30^∗^α (see **Figure 4D**).

A subsequent visual examination of the topographic distributions of the magnetic field was performed to confirm the results of the TMP analysis. Over time, the topographies revealed the three characteristic phases of engagement, stability, and disengagement for an IPS around 0.50 and 1.00^∗^α, and an unstable behavior for 1.30–2.30^∗^α. Thus, the visual analysis of the topographies confirmed the performed TMP analysis as a suitable method for characterizing topographic behavior. The classification results of the analyses in the spatial domain were in line with our findings in the frequency domain in terms of frequency selectivity (see **Figure 3C**).

### Time Domain

**FIGURE 5:**
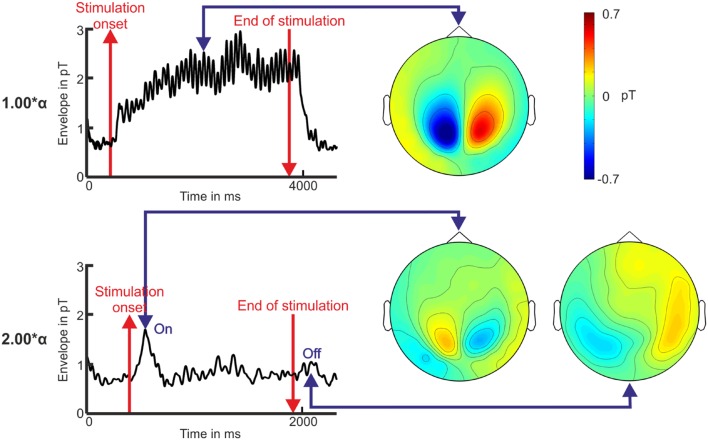
**Envelope over time and topography of the magnetometer signals illustrating the photic driving effect at 1.00^∗^α and the on-/off-response for the stimulation with 2.00^∗^α for volunteer P10.** Baseline is displayed 500 ms before the stimulation onset and 1 s after the end of the stimulation (red lines). The blue arrows mark the time points that the topographies display.

Resonance phenomena for stimulation frequencies at or close to 2.0^∗^α did not appear, neither was frequency entrainment observed (see **Figure 2** for time domain, **Figure 3** for frequency domain, and **Figure 4** for spatial domain). We found a noticeable brief rise in amplitude in the square root of the MGFP as well as in the envelopes shortly after the onset of the IPS. An example of the calculated envelopes for stimulation at 1.00 and at 2.00^∗^α is presented in **Figure 5.** The rise in amplitude is highlighted in **Figure 5** by the first blue arrow. A similar rise in amplitude, although smaller and less distinct than the one at the beginning of the stimulation, occurred shortly after the end of the stimulation for several stimulation frequencies (**Figure 5**, second blue arrow). Across all stimulation frequencies, the on-response was visible in 8 out of 12 volunteers, while the off-response was visible in 5 out of 12 volunteers.

The topographies show similar characteristics for the photic driving at 1.00^∗^α, consisting of alpha frequency entrainment as well as resonance phenomena, and the detected on- and off-response of the 2^∗^α stimulation (see **Figure 5**). Because not every volunteer showed an on-response, statistics on the on- and off-responses were based on eight participants per stimulation frequency. The on-response appeared with a latency of 201.7 ± 18.1 ms after the onset of the stimulation. No statistically significant correlations between latency, amplitude, stimulation frequency, and stimulation frequency sequence were found by ANOVA [e.g., *H*_0_ for stimulation frequency dependency: μ_1_(*f*_1_) = μ_2_(*f*_2_) = … = μ_20_(*f*_20_); *p* > 0.05] on the on-responses. We found the off-responses (153.43 ± 65.06 ms) to appear more often for high stimulation frequencies (1.30–2.30^∗^α) than for low stimulation frequencies (0.40–1.10^∗^α) [*t*-test; *H*_0_: μ_1_(*f*_1,_ …, *f*_12_) = μ_2_(*f*_13,_ …, *f*_20_); *p* < 0.05). As seen in **Figure 5**, the topographic distributions of the off-response and the on-response revealed a related dipolar pattern.

## Discussion

To the best of our knowledge, this is the first study to provide evidence for rod-input driven frequency entrainment and resonance effects in a photic driving experiment. Two main findings support our argument: (i) we found resonance phenomena and frequency entrainment for stimulation frequencies up to 1.30^∗^α and no resonance or entrainment above this frequency, which can be explained with the temporal properties of rod pathway; (ii) we found an on- and an off-response in the recordings for frequencies above 1.30^∗^α indicating that volunteers perceived a constant stimulation instead of a flicker stimulation.

In this study, radiometric measurement ensured well defined scotopic vision in the human eye during the photic driving experiment, which is dominated by rods. Rod photoreceptors process visual information considerably slower compared to cone photoreceptors (Sharpe et al., 1989; Stockman et al., 1995). The individual flicker fusion threshold, which affects the response behavior of the volunteer’s visual system, is influenced by the light intensity of the photic stimulus, as well as the adaption condition of the eye (Wiemeyer, 1990). Consequently, a rod-driven oscillatory response caused by a periodic input would be limited to a stimulation frequency of approximately 15 Hz (Stockman et al., 1991). By contrast, a cone driven input led to resonance phenomena for stimulation at frequencies up to 90 Hz (Herrmann, 2001). We observed no resonance phenomena for stimulation frequencies higher than 1.10^∗^α, which equals 11.5 ± 1.2 Hz in our study population. Additionally, frequency entrainment was not found for stimulation frequencies above 1.30^∗^α, which equals 13.6 ± 1.4 Hz. This finding indicates that photic driving under scotopic conditions is limited by the flicker perception range of the rod photoreceptors. The alpha desynchronization for flicker frequencies above 1.30^∗^α, as observed in **Figure 3**, might also be explained by the reduction of rhythmic brain activity by visual stimuli (Vijn et al., 1991), following the concept of event-related synchronization and desynchronization of the visual cortex, see (Pfurtscheller Lopes and da Silva, 1999) for an overview.

Our study provides latency and topography information for the visually evoked field (VEF) related to the onset and termination of a scotopic flickering visual stimulus (on-response and off-response). The on-responses in our recordings occurred in a narrow range of latencies around 201.7 ± 18.15 ms (mean ± SD) for all stimulation frequencies. This on-response characterizes the first perception of a visual stimulus. The latencies are compatible with ERG recordings, where single flash waveforms are the first response to a flickering stimulus (Marmor et al., 2009), and with visually evoked potentials (VEP) produced by single stimuli in scotopic vision (Schlegelmilch, 2004). The intra-individually stable topographies over the head at on-response latency support our assumption of an initial VEF.

Our analysis further revealed an increase in amplitude shortly after the end of the IPS. As this effect occurred significantly more often for higher stimulation frequencies (1.30–2.30^∗^α) than for lower stimulation frequencies (0.40–1.10^∗^α), it can be interpreted as a result of exceeding of the rod-dominated individual flicker fusion threshold. Assuming that the volunteers perceived the flicker light as a static illumination when the flicker fusion threshold is exceeded, an off-response can be expected in the MEG (Clynes et al., 1964). Therefore, the occurrence of an on-response as well as an off-response as transient responses toward flicker stimulation at frequencies ≥ 1.30^∗^α may be indicative of perception via rods in our experiment. Furthermore, this might explain the comparatively lower alpha rhythm activity for IPS ≥ 1.30^∗^α because the visual stimuli are no longer perceived as flickering light. This could indicate an event-related desynchronization of the underlying neural network (Pfurtscheller Lopes and da Silva, 1999).

We found strong alpha resonance phenomena for an IPS at stimulation frequencies around the volunteer’s alpha frequency (0.95–1.10^∗^α) and the first subharmonic (0.50–0.55^∗^α). This confirmed the results of our previous analysis (Schwab et al., 2006). The topographic engagement and disengagement process for the underlying neural network was described by correlation coefficient sequences between an individual reference TMP atom and TMP atoms for each stimulation frequency. In those sequences, three *phases* were found characterizing the engagement, plateau, and disengagement. The preservation of the photic driving effect shortly after the end of the IPS is in line with earlier observations (Halbleib et al., 2012; Spaak et al., 2014).

A comparison with the former results of Halbleib et al. (2012) revealed a less stable *plateau phase* in our correlation coefficient sequences meaning that the coefficients appeared to have a higher variance. This may be explained by the fact that we used whole head recordings in contrast to the previously used occipital sensor setup with 31 channels. In whole head data, the occipital region has less weighted influence on the selection of the TMP atom, since a higher number and wider spread channels are considered (Gratkowski et al., 2007). Besides, TMP was found to be powerful tool for summarizing the spatiotemporal behavior of MEG measurements.

On average, frequency entrainment was observed for 7 out of 12 volunteers for stimulation frequencies close to the individual alpha frequency or close to half of the individual alpha frequency (0.40, 0.45, 0.55, 0.60, 0.90, 0.95, 1.05, and 1.10^∗^α). Ten out of twelve volunteers showed a resonant response for 1.00^∗^α. Other studies reported driving responses of 50–80% for healthy volunteers depending on the presented frequency (Lazarev et al., 2004; Miranda de Sá and Infantosi, 2005), which is in good agreement with the results in our study. The variability of the found responses can be explained by the variability of the individual alpha rhythm itself (Garn et al., 2012). The alpha rhythm is among others related to age, memory performance, and the mental state of the volunteer (Klimesch, 1996), yielding large inter- and intra-individual differences in the steady-state responses (Pigeau and Frame, 1992). Moreover, the one-time estimation of the individual alpha rhythm at the beginning of each measurement session did not capture the temporal change of the volunteer’s alpha activity during the measurements. All these factors contribute to the variability seen in the entrainment classification and the alpha ratios (**Figure 3D**) and the variability in the topographic correlation coefficient sequences (**Figures 4C,D**).

Frequency entrainment and resonance produced by IPS can be explained by a neural oscillator network (Mundy-Castle, 1953; Kawaguchi et al., 1993). An alternative explanation has been provided by Capilla et al. (2011), who proposed the concept of a temporal superposition of transient event-related responses. However, recent evidence presented by Notbohm et al. (2016) indicates that entrainment but not superposition of event-related responses to be associated with steady-state visual evoked potentials.

The dynamics of the alpha frequency entrainment process and those of accompanying phase-locked gamma oscillations (30–60 Hz) were investigated by Wacker et al. (2011) using the recordings of Schwab et al. (2006) for the stimulation frequencies 0.80, 1.05, and 1.20^∗^α. A frequency entrainment is observed within the first second after stimulus onset, accompanied by an extensively rising phase-locking to the onset (400 ms after onset). Resonance appears approximately 500 ms after frequency entrainment, indicating that resonance and frequency entrainment do not occur simultaneously, which is consistent with our observations. Wacker et al. (2011) found that a certain degree of frequency entrainment remained effective during the rest periods of 4 s between the stimulation trains of a flicker frequency. Besides an IPS driven oscillation, the findings of Wacker et al. (2011) indicated sustained generator activation during flicker stimulation.

The frequency for which the maximum resonance in photic driving experiments occurs is controversial. For example Herrmann (2001) and Pastor et al. (2003) found the strongest responses on average to flicker stimuli of ∼15 Hz, whereas in other experiments (Lazarev et al., 2001; Schwab et al., 2006) the strongest resonance occurred at flicker frequencies closest to the individual alpha frequency (∼10 Hz). The perception performance of the human eye depends on the stimulation conditions (flicker light intensity, ambient light, adaption of the human eye, and other factors) and thereby varies over the disparate experimental setups. Our study showed that rod-driven photic driving is limited by the flicker fusion threshold. Resonance phenomena caused by photic driving were modeled by Spiegler et al. (2011) using the concept of a periodically forced oscillator (Janson and Rit neural mass model). In that study, the (non-linear) dynamics of the system are dependent on the relationship between flicker frequency and intrinsic frequency, as well as on the intensity of the stimulus. Our investigations support those dependencies by demonstrating frequency selectivity of the underlying network as well as an impact of the presented stimulus intensity.

In summary, we propose the existence of frequency entrainment and resonance phenomena in rod-driven photic driving experiments. It is limited by the flicker fusion threshold at around 15 Hz. Photometrical measurements of stimulation setups for IPS are essential to predict the response behavior. Future studies are required to perform direct comparisons between rod and cone driven responses.

## Author Contributions

DS, SK, KS, HW, and JH designed the study. CS, DS, SK, DJ, and JH analyzed the data. All authors wrote and reviewed the paper.

## Conflict of Interest Statement

The authors declare that the research was conducted in the absence of any commercial or financial relationships that could be construed as a potential conflict of interest.

## References

[B1] Başar E. Schürmann M. Başar-Eroglu C. Karakaş S. (1997). Alpha oscillations in brain functioning: an integrative theory. *Int. J. Psychophysiol.* 26 5–29. 10.1016/S0167-8760(97)00753-8 9202992

[B2] Bierman A. Figueiro M. G. Rea M. S. (2011). Measuring and predicting eyelid spectral transmittance. *J. Biomed. Opt.* 16:67011 10.1117/1.3593151 21721832

[B3] Capilla A. Pazo-Alvarez P. Darriba A. Campo P. Gross J. (2011). Steady-state visual evoked potentials can be explained by temporal superposition of transient event-related responses. *PLoS ONE* 6:e14543 10.1371/journal.pone.0014543 PMC3022588 21267081

[B4] Clynes M. Kohn M. Lifshitz K. (1964). Dynamics of light evoked potentials, their modification under hypnosis, and on-line correlation in relation to rhythmic components. *Ann. N. Y. Acad. Sci.* 112 468–509. 10.1111/j.1749-6632.1964.tb26764.x 14188111

[B5] Drake M. E. Jr. Shy K. E. Liss L. (1989). Quantitation of photic driving in dementia with normal EEG. *Clin. Electroencephalogr.* 20 153–155. 10.1177/155005948902000307 2752585

[B6] Fedotchev A. I. Bondar A. T. Konovalov V. F. (1990). Stability of resonance EEG reactions to flickering light in humans. *Int. J. Psychophysiol.* 9 189–193. 10.1016/0167-8760(90)90073-M 2228753

[B7] Fukami T. Ishikawa F. Ishikawa B. Saito Y. (2008). Quantitative evaluation of photic driving response for computer-aided diagnosis. *J. Neural Eng.* 5 411–421. 10.1088/1741-2560/5/4/006 18971516

[B8] Garn H. Waser M. Lechner M. Dorfer M. Grossegger D. (2012). “Robust, automatic real-time monitoring of the time course of the individual alpha frequency in the time and frequency domain,” in *Proceedings of the Engineering in Medicine and Biology Society (EMBC), 34th Annual International Conference of the IEEE*, (San Diego, CA: IEEE, 2012), 2227–2231. 10.1109/EMBC.2012.6346405 23366366

[B9] Gebber G. L. Zhong S. Lewis C. Barman S. M. (1999). Human brain alpha rhythm: nonlinear oscillation or filtered noise? *Brain Res.* 818 556–560. 10.1016/S0006-8993(98)01303-1 10082847

[B10] Gratkowski M. Haueisen J. Arendt-Nielsen L. Zanow F. (2007). Topographic Matching Pursuit of spatio-temporal bioelectromagnetic data. *Przegl. Elektrotech.* 83 138–141.

[B11] Halbleib A. Gratkowski M. Schwab K. Ligges C. Witte H. Haueisen J. (2012). Topographic analysis of engagement and disengagement of neural oscillators in photic driving: a combined EGG/MEG study. *J. Clin. Neurophysiol.* 29 33–41. 10.1097/WNP.0b013e318246ad6e 22353983

[B12] Hayashi C. (1985). *Nonlinear Oscillations in Physical Systems.* Princeton, NJ: Princeton Univ Press.

[B13] Herrmann C. S. (2001). Human EEG responses to 1-100 Hz flicker: resonance phenomena in visual cortex and their potential correlation to cognitive phenomena. *Exp. Brain Res.* 137 346–353. 10.1007/s002210100682 11355381

[B14] Hess R. F. Nordby K. (1986). Spatial and temporal properties of human rod vision in the achromat. *J. Physiol.* 371 387–406. 10.1113/jphysiol.1986.sp015982 3486272 PMC1192731

[B15] Kalitzin S. Parra J. Velis D. N. Lopes da Silva F. H. (2002). Enhancement of phase clustering in the EEG/MEG gamma frequency band anticipates transitions to paroxysmal epileptiform activity in epileptic patients with known visual sensitivity. *IEEE Trans. Biomed. Eng.* 49 1279–1286. 10.1109/TBME.2002.804593 12450358

[B16] Kawaguchi T. Jijiwa H. Watanabe S. (1993). The dynamics of phase relationships of alpha waves during photic driving. *Electroencephalogr. Clin. Neurophysiol.* 87 88–96. 10.1016/0013-4694(93)90115-C 7691545

[B17] Klimesch W. (1996). Memory processes, brain oscillations and EEG synchronization. *Int. J. Psychophysiol.* 24 61–100. 10.1016/S0167-8760(96)00057-8 8978436

[B18] Lazarev V. V. Infantosi A. F. C. Valencio-de-Campos D. de Azevedo L. C. (2004). Topographic aspects of photic driving in the electroencephalogram of children and adolescents. *Braz. J. Med. Biol. Res.* 37 879–891. 10.1590/S0100-879X2004000600014 15264032

[B19] Lazarev V. V. Simpson D. M. Schubsky B. M. de Azevedo L. C. (2001). Photic driving in the electroencephalogram of children and adolescents: harmonic structure and relation to the resting state. *Braz. J. Med. Biol. Res.* 34 1573–1584. 10.1590/S0100-879X2001001200010 11717711

[B20] Lopes da Silva F. H. (1991). Neural mechanisms underlying brain waves: from neural membranes to networks. *Electroencephalogr. Clin. Neurophysiol.* 79 81–93. 10.1016/0013-4694(91)90044-5 1713832

[B21] Mallat S. G. Zhang Z. (1993). Matching pursuit with time-frequency dictionaries. *IEEE Trans. Signal Process.* 41 3397–3415. 10.1109/78.258082

[B22] Marmor M. F. Fulton A. B. Holder G. E. Miyake Y. Brigell M. Bach M. (2009). ISCEV standard for full-field clinical electroretinography (2008 update). *Doc. Ophthalmol.* 118 69–77. 10.1007/s10633-008-9155-4 19030905

[B23] Miranda de Sá A. M. F. L. Infantosi A. F. C. (2005). Evaluating the entrainment of the alpha rhythm during stroboscopic flash stimulation by means of coherence analysis. *Med. Eng. Phys.* 27 167–173. 10.1016/j.medengphy.2004.09.011 15642512

[B24] Mundy-Castle A. C. (1953). An analysis of central responses to photic stimulation in normal adults. *Electroencephalogr. Clin. Neurophysiol.* 5 1–22. 10.1016/0013-4694(53)90048-0 13033804

[B25] Notbohm A. Kurths J. Herrmann C. S. (2016). Modification of brain oscillations via rhythmic light stimulation provides evidence for entrainment but not for superposition of event-related responses. *Front. Hum. Neurosci.* 10:10 10.3389/fnhum.2016.00010 PMC4737907 26869898

[B26] Pastor M. A. Artieda J. Arbizu J. Valencia M. Masdeu J. C. (2003). Human cerebral activation during steady-state visual-evoked responses. *Int. J. Neurosci.* 23 11621–11627. 10.1523/JNEUROSCI.23-37-11621.2003 PMC6740964 14684864

[B27] Pfurtscheller G. Lopes da Silva F. H. (1999). Event-related EEG/MEG synchronization and desynchronization: basic principles. *Clin. Neurophysiol.* 110 1842–1857. 10.1016/S1388-2457(99)00141-8 10576479

[B28] Pigeau R. A. Frame A. M. (1992). Steady-state visual evoked responses in high and low alpha subjects. *Electroencephalogr. Clin. Neurophysiol.* 84 101–109. 10.1016/0168-5597(92)90014-3 1372224

[B29] Pikovsky A. Rosenblum M. Kurths J. (2001). *Synchronization: A Universal Concept in Nonlinear Sciences.* Cambridge: Cambridge University Press.

[B30] Regan D. (1965). Some characteristics of average steady-state and transient responses evoked by modulated light. *Electroencephalogr. Clin. Neurophysiol.* 20 238–248. 10.1016/0013-4694(66)90088-5 4160391

[B31] Sakamoto H. Inouye T. Shinosaki K. (1993). Preservation of alpha rhythm shortly after photic driving. *Int. J. Neurosci.* 73 227–233. 10.3109/00207459308986673 8169058

[B32] Schlegelmilch F. (2004). *Methodische und technisch-experimentelle Untersuchungen zur Realisierung einer elektrophysiologischen Blaukanalstimulation*. Ph.D. thesis, Ilmenau University of Technology, Ilmenau.

[B33] Schwab K. Ligges C. Jungmann T. Hilgenfeld B. Haueisen J. Witte H. (2006). Alpha entrainment in human electroencephalogram and magnetoencephalogram recordings. *Neuroreport* 17 1829–1833. 10.1097/01.wnr.0000246326.89308.ec 17164673

[B34] Sharpe L. T. Stockman A. MacLeod D. I. A. (1989). Rod flicker perception: scotopic duality, phase lags and destructive interference. *Vision Res.* 29 1539–1559. 10.1016/0042-6989(89)90137-5 2635479

[B35] Silberstein R. B. (1995). “Steady-state visually evoked potentials, brain resonances, and cognitive processes,” in *Neocortical Dynamics and Human EEG Rhythms*, ed. Nunez P. L. (Oxford: Oxford University Press).

[B36] Spaak E. de Lange F. P. Jensen O. (2014). Local Entrainment of Alpha oscillations by visual stimuli causes cyclic modulation of perception. *J. Neurosci.* 34 3536–3544. 10.1523/JNEUROSCI.4385-13.2014 24599454 PMC6608988

[B37] Spiegler A. Knösche T. R. Schwab K. Haueisen J. Atay F. M. (2011). Modeling brain resonance phenomena using a neural mass mode. *PLoS Comput. Biol.* 7:e1002298 10.1371/journal.pcbi.1002298 PMC3245303 22215992

[B38] Stam C. J. Pijn J. P. M. Suffczynski P. Lopes da Silva F. H. (1999). Dynamics of the human alpha rhythm: evidence for non-linearity? *Clin. Neurophysiol.* 110 1801–1813. 10.1016/S1388-2457(99)00099-1 10574295

[B39] Stockman A. Sharpe L. T. (2006). Into the twilight zone: the complexities of mesopic vision and luminous efficiency. *Ophthalmic. Physiol. Opt.* 26 225–239. 10.1111/j.1475-1313.2006.00325.x 16684149

[B40] Stockman A. Sharpe L. T. Rüther K. Nordby K. (1995). Two signals in the human rod visual system: a model based on electrophysiological data. *Vis. Neurosci.* 12 951–970. 10.1017/S0952523800009500 8924418

[B41] Stockman A. Sharpe L. T. Zrenner E. Nordby K. (1991). Slow and fast pathways in the human rod visual system: electrophysiology and psychophysics. *J. Opt. Soc. Am. A* 8 1657–1665. 10.1364/JOSAA.8.001657 1941296

[B42] Stough C. Donaldson C. Scarlata B. Ciorciari J. (2001). Psychophysiological correlates of the NEO PI-R openness, agreeableness and conscientiousness: preliminary results. *Int. J. Psychophysiol.* 41 87–91. 10.1016/S0167-8760(00)00176-8 11239700

[B43] Takahashi T. (2005). “Activation methods,” in *Electroencephalography: Basic Principles, Clinical Applications, and Related Fields*, eds Niedermeyer E. da Silva F. H. (Philadelphia, PA: Lippincott Williams&Wilkins), 281–304.

[B44] Tovée M. J. (1996). *An Introduction to the Visual System.* Cambridge: Cambridge University Press.

[B45] Van der Tweel L. H. Verduyn Lunel H. F. E. (1964). Human visual responses to sinusoidally modulated light. *Electroencephalogr. Clin. Neurophysiol.* 18 587–598. 10.1016/0013-4694(65)90076-3 14296836

[B46] Vijn P. C. M. van Dijk B. W. Spekreijse H. (1991). Visual stimulation reduces EEG activity in man. *Brain Res.* 550 49–53. 10.1016/0006-8993(91)90403-I 1889000

[B47] Wacker M. Galicki M. Putsche P. Milde T. Schwab K. Haueisen J. (2011). A time-variant processing approach for the analysis of alpha and gamma MEG oscillations during flicker stimulus generated entrainment. *IEEE Trans. Biomed. Eng.* 58 3069–3077. 10.1109/TBME.2011.2160640 21712153

[B48] Wiemeyer J. (1990). *Zentralnervöse Aktivierung und Sportliche Leistung.* Köln: Verlag Sport und Buch.

