# Supplementary material for: Rod Driven Frequency Entrainment and Resonance Phenomena
Source: Front Hum Neurosci. 2016 Aug 18;10:413. doi: 10.3389/fnhum.2016.00413 (PMC4989477; doi:10.3389/fnhum.2016.00413)
Supplement: Supplementary file 1 [file Data_Sheet_1.DOCX]

Supplementary Material

Rod Driven Frequency Entrainment and Resonance Phenomena

Christina Salchow, Daniel Strohmeier, Sascha Klee, Dunja Jannek, Karin Schiecke, Herbert Witte, Arye Nehorai, Jens Haueisen*

*** Correspondence:** Jens Haueisen: jens.haueisen@tu-ilmenau.de

**1. Supplementary figures: Individual results in the frequency domain**


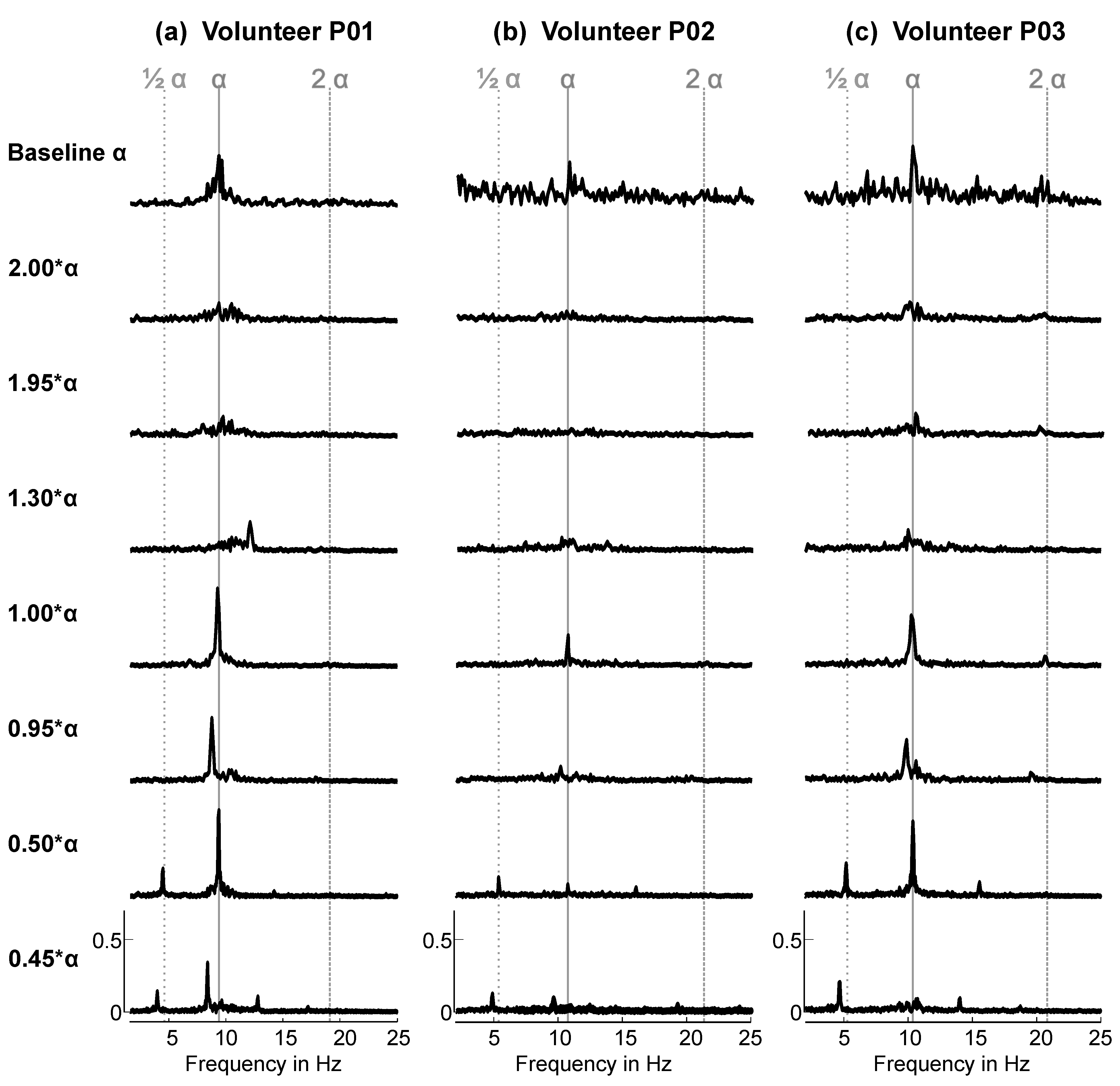


**Figure S1.** Amplitude spectra for seven stimulation frequencies and resting MEG (resting state α) for **(a)** volunteer P01, **(b)** volunteer P02, and **(c)** volunteer P03. The amplitude spectra (pT∙cm^-1^) are shown as an average over 24 occipital gradiometer channels and are displayed in the frequency range from 2–25 Hz. The estimated individual alpha frequency was 9.6 Hz for volunteer P01, 10.7 Hz for volunteer P02, and 10.4 Hz for volunteer P03.

**
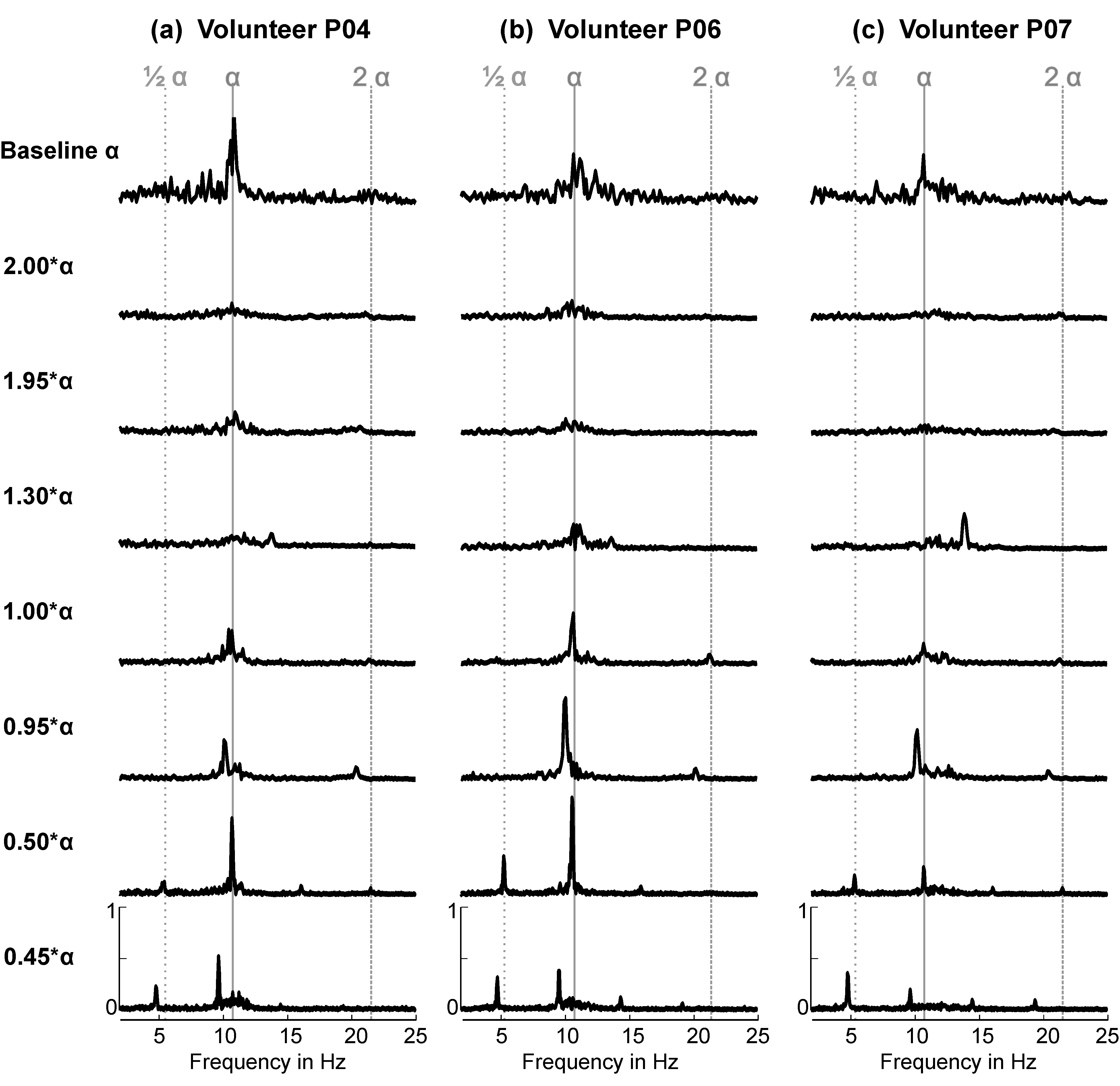
**

**Figure S2.** Amplitude spectra for seven stimulation frequencies and resting MEG (resting state α) for **(a)** volunteer P04, **(b)** volunteer P06, and **(c)** volunteer P07. The amplitude spectra (pT∙cm^-1^) are shown as an average over 24 occipital gradiometer channels and are displayed in the frequency range from 2–25 Hz. The estimated individual alpha frequency was 10.8 Hz for volunteer P04, 10.7 Hz for volunteer P06, and 10.8 Hz for volunteer P07.


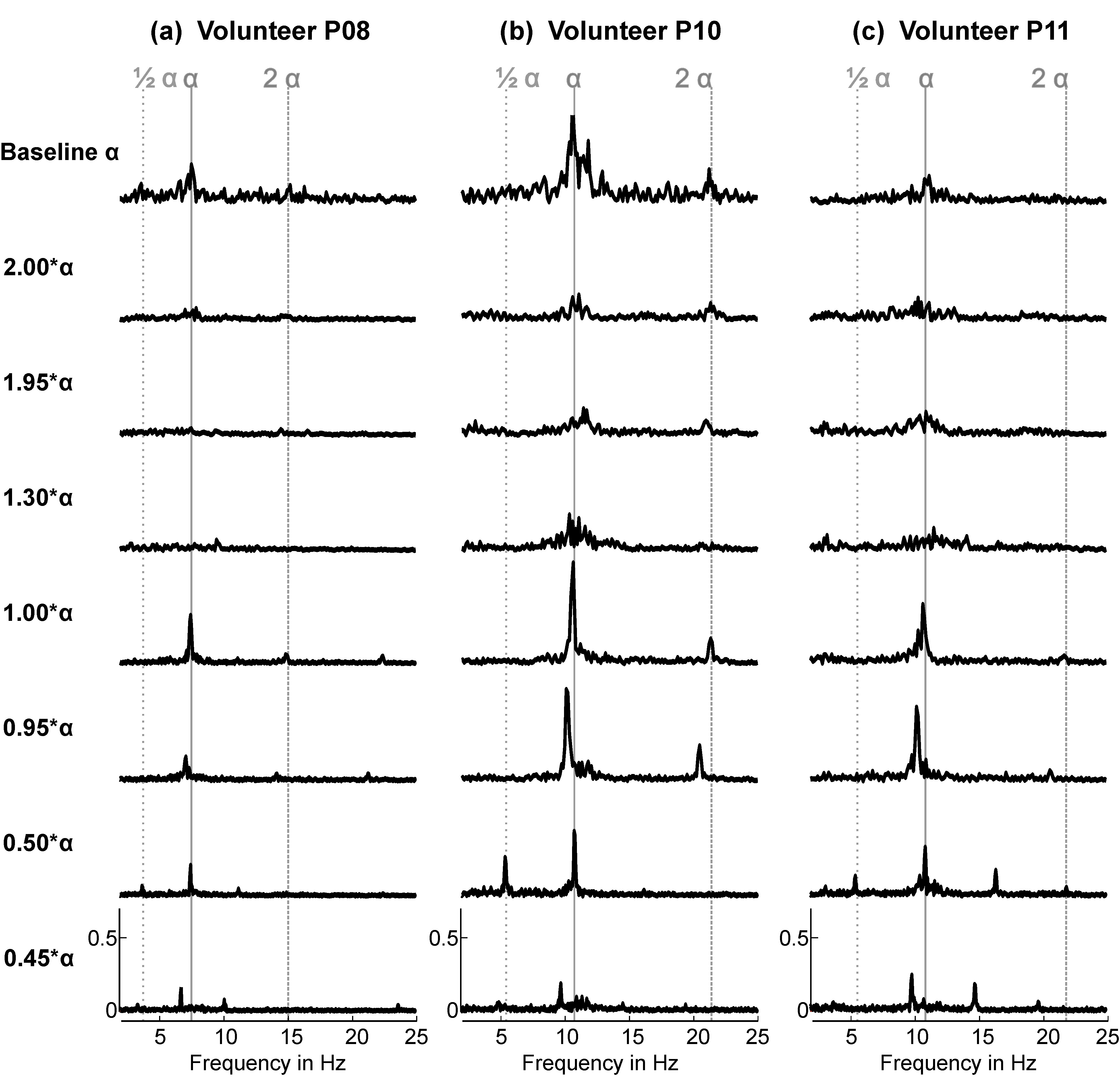


**Figure S3.** Amplitude spectra for seven stimulation frequencies and resting MEG (resting state α) for **(a)** volunteer P08, **(b)** volunteer P10, and **(c)** volunteer P11. The amplitude spectra (pT∙cm^-1^) are shown as an average over 24 occipital gradiometer channels and are displayed in the frequency range from 2–25 Hz. The estimated individual alpha frequency was 7.5 Hz for volunteer P08, 10.8 Hz for volunteer P10, and 11 Hz for volunteer P11.

**
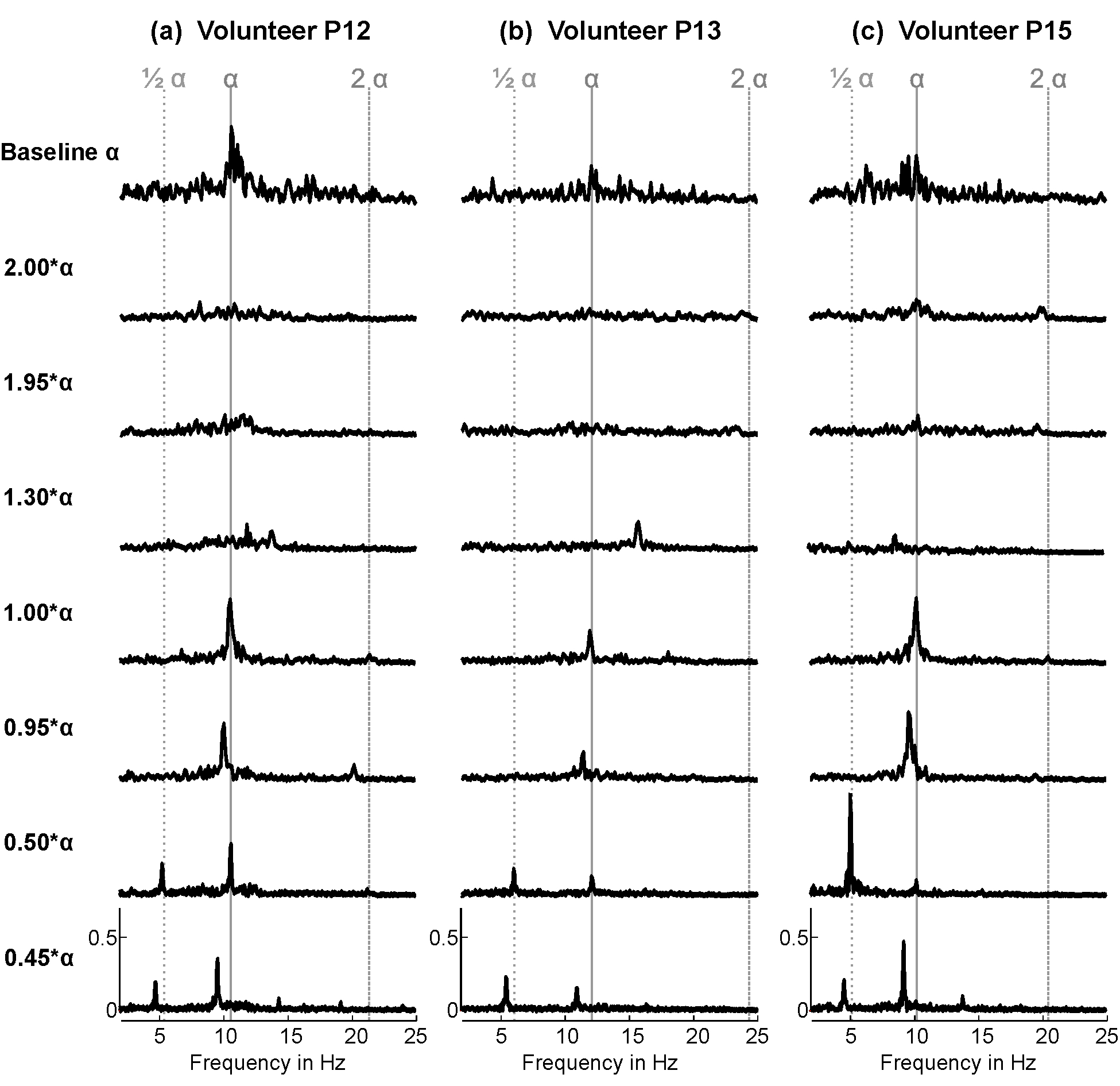
**

**Figure S4.** Amplitude spectra for seven stimulation frequencies and resting MEG (resting state α) for **(a)** volunteer P12, **(b)** volunteer P13, and **(c)** volunteer P15. The amplitude spectra (pT∙cm^-1^) are shown as an average over 24 occipital gradiometer channels and are displayed in the frequency range from 2–25 Hz. The estimated individual alpha frequency was 10.7 Hz for volunteer P12, 12.2 Hz for volunteer P13, and 10.3 Hz for volunteer P15.
